# Mapping ICD-10 and ICD-10-CM Codes to Phecodes: Workflow Development and Initial Evaluation

**DOI:** 10.2196/14325

**Published:** 2019-11-29

**Authors:** Patrick Wu, Aliya Gifford, Xiangrui Meng, Xue Li, Harry Campbell, Tim Varley, Juan Zhao, Robert Carroll, Lisa Bastarache, Joshua C Denny, Evropi Theodoratou, Wei-Qi Wei

**Affiliations:** 1 Department of Biomedical Informatics Vanderbilt University Medical Center Nashville, TN United States; 2 Medical Scientist Training Program Vanderbilt University School of Medicine Nashville, TN United States; 3 Centre for Global Health Research Usher Institute of Population Health Sciences and Informatics The University of Edinburgh Edinburgh United Kingdom; 4 Public Health and Intelligence Strategic Business Unit National Services Scotland Edinburgh United Kingdom; 5 Department of Medicine Vanderbilt University Medical Center Nashville, TN United States; 6 Edinburgh Cancer Research Centre Institute of Genetics and Molecular Medicine University of Edinburgh Edinburgh United Kingdom

**Keywords:** electronic health record, genome-wide association study, phenome-wide association study, phenotyping, medical informatics applications, data science

## Abstract

**Background:**

The phecode system was built upon the International Classification of Diseases, Ninth Revision, Clinical Modification (ICD-9-CM) for phenome-wide association studies (PheWAS) using the electronic health record (EHR).

**Objective:**

The goal of this paper was to develop and perform an initial evaluation of maps from the International Classification of Diseases, 10th Revision (ICD-10) and the International Classification of Diseases, 10th Revision, Clinical Modification (ICD-10-CM) codes to phecodes.

**Methods:**

We mapped ICD-10 and ICD-10-CM codes to phecodes using a number of methods and resources, such as concept relationships and explicit mappings from the Centers for Medicare & Medicaid Services, the Unified Medical Language System, Observational Health Data Sciences and Informatics, Systematized Nomenclature of Medicine-Clinical Terms, and the National Library of Medicine. We assessed the coverage of the maps in two databases: Vanderbilt University Medical Center (VUMC) using ICD-10-CM and the UK Biobank (UKBB) using ICD-10. We assessed the fidelity of the ICD-10-CM map in comparison to the gold-standard ICD-9-CM phecode map by investigating phenotype reproducibility and conducting a PheWAS.

**Results:**

We mapped >75% of ICD-10 and ICD-10-CM codes to phecodes. Of the unique codes observed in the UKBB (ICD-10) and VUMC (ICD-10-CM) cohorts, >90% were mapped to phecodes. We observed 70-75% reproducibility for chronic diseases and <10% for an acute disease for phenotypes sourced from the ICD-10-CM phecode map. Using the ICD-9-CM and ICD-10-CM maps, we conducted a PheWAS with a Lipoprotein(a) genetic variant, rs10455872, which replicated two known genotype-phenotype associations with similar effect sizes: coronary atherosclerosis (ICD-9-CM: *P*<.001; odds ratio (OR) 1.60 [95% CI 1.43-1.80] vs ICD-10-CM: *P*<.001; OR 1.60 [95% CI 1.43-1.80]) and chronic ischemic heart disease (ICD-9-CM: *P*<.001; OR 1.56 [95% CI 1.35-1.79] vs ICD-10-CM: *P*<.001; OR 1.47 [95% CI 1.22-1.77]).

**Conclusions:**

This study introduces the beta versions of ICD-10 and ICD-10-CM to phecode maps that enable researchers to leverage accumulated ICD-10 and ICD-10-CM data for PheWAS in the EHR.

## Introduction

### Background

Electronic health records (EHRs) have become a powerful resource for biomedical research in the last decade, and many studies based on EHR data have used International Classification of Diseases (ICD) codes [1]. When linked to DNA biobanks, healthcare information in EHRs can be a tool to help discover genetic associations by using billing codes in phenotyping algorithms. The phenome-wide association study (PheWAS) paradigm was introduced in 2010 as an approach that scans across a range of phenotypes, similar to what is done for the genome in genome-wide association studies. Studies using PheWAS have replicated hundreds of known genotype-phenotype associations and discovered dozens of new ones [2-12]. The initial version of phecodes consisted of 733 custom groups of ICD Ninth Revision, Clinical Modification (ICD-9-CM) diagnosis codes. The most recent iteration of phecodes consists of 1866 hierarchical phenotype codes that map to 15,558 ICD-9-CM codes [13,14]. However, many health systems and international groups use the International Classification of Diseases, 10th Revision (ICD-10) or the International Classification of Diseases, 10th Revision, Clinical Modification (ICD-10-CM) codes [15], therefore necessitating a new phecode map.

### Transition from ICD-9 to ICD-10

In 1979, the World Health Organization (WHO) developed ICD-9 to track mortality and morbidity. To improve its application to clinical billing, the United States National Center for Health Statistics (NCHS) modified ICD-9 codes to create ICD-9-CM, whose end-of-life date was scheduled around the year 2000 but was delayed until October 2015 [15]. In 1990, the WHO developed ICD-10 [16], which the NCHS used to create ICD-10-CM to replace ICD-9-CM.

Moving from ICD-9-CM to ICD-10-CM led to major structural changes in the coding system. First, the structure moved from a broadly numeric-based system in ICD-9-CM (eg, 474.11 for “Hypertrophy of tonsils alone”) to an alphanumeric system in ICD-10-CM (eg, J35.1 for the same condition). Second, ICD-10-CM contains much more granular information than ICD-9-CM, as seen with the approximately tenfold increase in the number of diabetes-related codes in ICD-10-CM. ICD-10-CM also differs from ICD-9-CM in terms of semantics and organization [15,17].

Compared to ICD-10, ICD-10-CM has even more codes and granularity. While the 2018AA Unified Medical Language System (UMLS) [18] contains 94,201 unique ICD-10-CM codes, it has 12,027 unique ICD-10 codes after exclusion of range codes (eg, ICD-10-CM A00-A09). Further, there are ICD-10 codes that do not exist in ICD-10-CM, and vice versa, like ICD-10 A16.9 “Respiratory tuberculosis unspecified, without mention of bacteriological or histological confirmation”, which has no ICD-10-CM equivalent. 

### Prior Work

To develop the original phecode system, one or more related ICD-9-CM codes were combined into distinct diseases or traits. For example, three depression-related ICD-9-CM codes, 311, 296.31, and 296.2, were condensed to phecode 296.2 “Depression”. With the help of clinical experts in disparate domains, such as cardiology and oncology, we have iteratively updated the phecode groupings [19].

The phecode scheme is unique because it has built-in exclusion criteria to prevent contamination by cases in the control cohort. This is an important feature, as case contamination of control groups decreases the statistical power for finding genotype-phenotype associations [20]. For each disease phenotype, we defined exclusion criteria by using our clinical knowledge and by consulting physician specialists.

An example for how users can use phecode exclusion criteria is illustrated by a type 2 diabetes study using EHRs. To define cases of type 2 diabetes, users include patients with ICD codes that map to phecode 250.2 “Type 2 diabetes”. To create the control cohort, they only include patients without phenotypes in the “Diabetes” group, which is comprised of phecodes in the range of 249-250.99. This prevents contamination of the control group by patients with diseases such as “Type 1 diabetes” (phecode 250.1) and “Secondary diabetes mellitus” (phecode 249). Excluded patients also include those with signs and symptoms commonly associated with type 2 diabetes, such as “Abnormal glucose” (phecode 250.4), which may indicate someone who has not yet been diagnosed with diabetes.

Though the phecode system is effective at replicating and identifying novel genotype-phenotype associations, PheWAS have largely been limited to using ICD-9-CM codes. A few studies have mapped ICD-10 codes to phecodes by converting ICD-10 to ICD-9-CM, and then mapping the converted ICD-9-CM codes to phecodes [3,10]. However, these studies limited their mappings to ICD-10 (non-CM) codes, did not provide a map to translate ICD-10-CM codes to phecodes, and did not evaluate the accuracy of these maps.

### Study Goals

In this study, we developed and evaluated maps of ICD-10 and ICD-10-CM codes to phecodes. The primary aims of this study were to create an initial beta map to perform PheWAS using ICD-10 and ICD-10-CM codes and to focus the analyses on PheWAS-relevant codes. Our goal was to demonstrate that researchers should expect similar results from the ICD-10-CM phecode map compared to the gold-standard ICD-9-CM map. To accomplish this goal, we investigated phecode coverage, phenotype reproducibility, and the results from a PheWAS.

## Methods

### Databases

In this study, we used data obtained from the Vanderbilt University Medical Center (VUMC) and UK Biobank (UKBB) databases. The VUMC EHR contains clinical information derived from the medical records of >3 million unique individuals. The UKBB is a prospective longitudinal cohort study designed to investigate the genetic and environmental determinants of diseases in UK adults. Between 2006-2010, the study recruited >500,000 men and women aged 40-69 years. Participants consented to allow their data to be linked to their medical records. EHR records from the UKBB were obtained under an approved data request application (ID:10775).

We used VUMC data with >2.5 years of ICD-10-CM data (October 10, 2015 to June 1, 2017) for inpatient and outpatient encounters. Comparatively, we used UKBB data with >2 decades of ICD-10 data [21] (April 1, 1995 to March 31, 2015) for only inpatient encounters.

### Mapping ICD-10-CM and ICD-10 Codes to Phecodes

**Figure 1:**
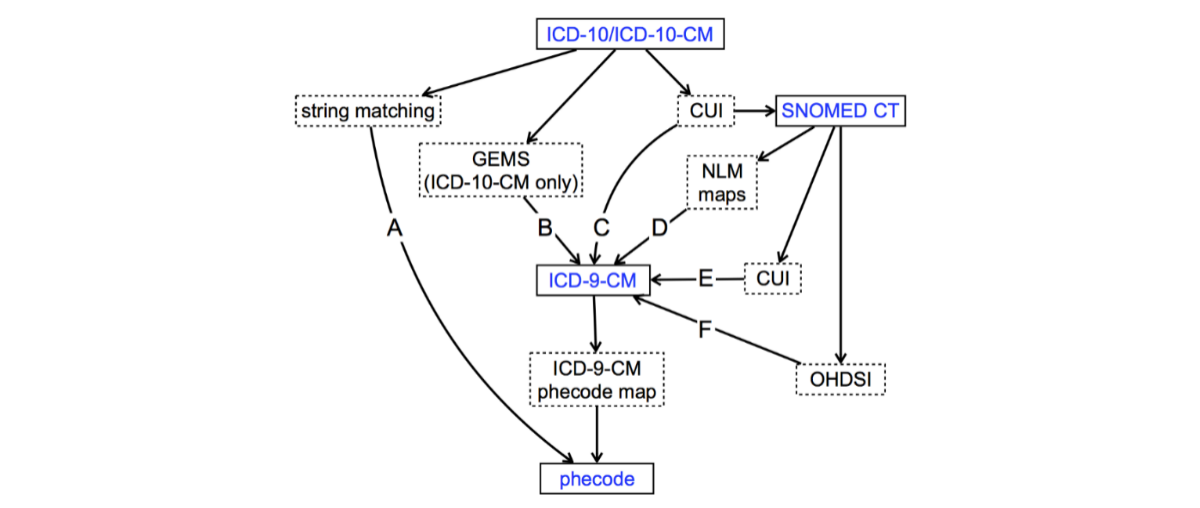
Mapping strategy for ICD-10 (non-CM) and ICD-10-CM diagnosis codes to phecodes. We mapped ICD-10-CM codes directly by matching code descriptions (path A) or indirectly to phecodes, using a number of manually validated mapping resources (paths B, C, D, E, and F). In path D, we used NLM’s SNOMED CT to create ICD-9-CM one-to-one and many-to-one maps [23]. To map ICD-9-CM codes to phecodes, we applied Phecode Map 1.2 with ICD-9 Codes (ICD-9-CM phecode map) [14]. Boxes with solid lines indicate clinical terminologies, and those with dashed lines describe the resources and mapping methods used. ICD-10-CM: International Classification of Diseases, Tenth Revision, Clinical Modification; CUI: Concept Unique Identifier; SNOMED CT: Systematized Nomenclature of Medicine Clinical Terms; GEMS: General Equivalence Mappings; NLM: National Library of Medicine; ICD-9-CM: International Classification of Diseases, Ninth Revision, Clinical Modification; OHDSI: Observational Health Data Sciences and Informatics.

We extracted ICD-10-CM codes from the 2018AA release of the UMLS [18] and used several automated methods to translate ICD-10-CM diagnosis codes to phecodes (Figure 1). We mapped 515 ICD-10-CM codes directly to phecodes by matching code descriptions regardless of capitalization (eg, ICD-10-CM H52.4 “Presbyopia” to phecode 367.4 “Presbyopia”). We mapped 82,287 ICD-10-CM codes indirectly to phecodes using the existing ICD-9-CM phecode map [14]. To convert ICD-10-CM codes indirectly to phecodes, we used General Equivalence Mappings (GEMS) provided by the Centers for Medicare & Medicaid Services that map ICD-10-CM to ICD-9-CM and vice versa [22]. We included both equivalent and nonequivalent GEMS mappings (ie, where the approximate flag was either 0 or 1). As an example of this indirect approach, to map ICD-10-CM E11.9 “Type 2 diabetes mellitus without complications” to phecode 250.2 “Type 2 diabetes,” we mapped ICD-10-CM E11.9 to ICD-9-CM 250.0 “Diabetes mellitus without mention of complication” to phecode 250.2.

Since the GEMS do not provide ICD-9-CM mappings for all ICD-10-CM codes [17], we complemented this approach with UMLS semantic mapping [24], Observational Health Data Sciences and Informatics (OHDSI) concept relationships [25,26], and National Library of Medicine (NLM) maps [23]. In this approach to indirect mapping, we first mapped ICD-10-CM codes to Systematized Nomenclature of Medicine Clinical Terms (SNOMED CT) through UMLS Concept Unique Identifier (CUI) equivalents, which were then converted to ICD-9-CM through either UMLS CUI equivalents [18,24], OHDSI [25], or NLM maps [23]. For example, we mapped ICD-10-CM L01.00 “Impetigo, unspecified” to CUI C0021099 to SNOMED CT 48277006 to OHDSI Concept ID 140480 to OHDSI Concept ID 44832600 to ICD-9-CM 684 and finally to phecode 686.2 “Impetigo”.

There were two general instances when an ICD-10-CM code mapped to more than one phecode. First, some ICD-10-CM codes mapped to both a parent phecode and one of its child phecodes that was lower in the hierarchy. To maintain the granular meanings of ICD-10-CM codes, we only kept the mappings to child phecodes, a decision that we could make due to the hierarchical structure of phecodes. For example, ICD-10-CM I10 “Essential (primary) hypertension” was mapped to phecodes 401 “Hypertension” and 401.1 “Essential hypertension”, but we only kept the mapping to phecode 401.1. Second, we kept all the mappings for ICD-10-CM codes that were translated to phecodes that were not in the same family. This can be seen in the mapping of ICD-10-CM D57.812 “Other sickle-cell disorders with splenic sequestration” to phecodes 282.5 “Sickle cell anemia” and 289.5 “Diseases of spleen”. This latter association created a polyhierarchical nature to phecodes that did not previously exist.

To map ICD-10 (non-CM) codes to phecodes, we also used ICD-10 codes from the 2018AA UMLS [18]. ICD-10 codes were mapped to phecodes in a similar manner to ICD-10-CM, but since a GEMS to translate ICD-10 to ICD-9-CM was not available, we used only string matching and previously manually reviewed resources from the UMLS [24], NLM [23], and OHDSI [25,26].

### Evaluation of Phecode Coverage of ICD-10 and ICD-10-CM in UKBB and VUMC

**Figure 2:**
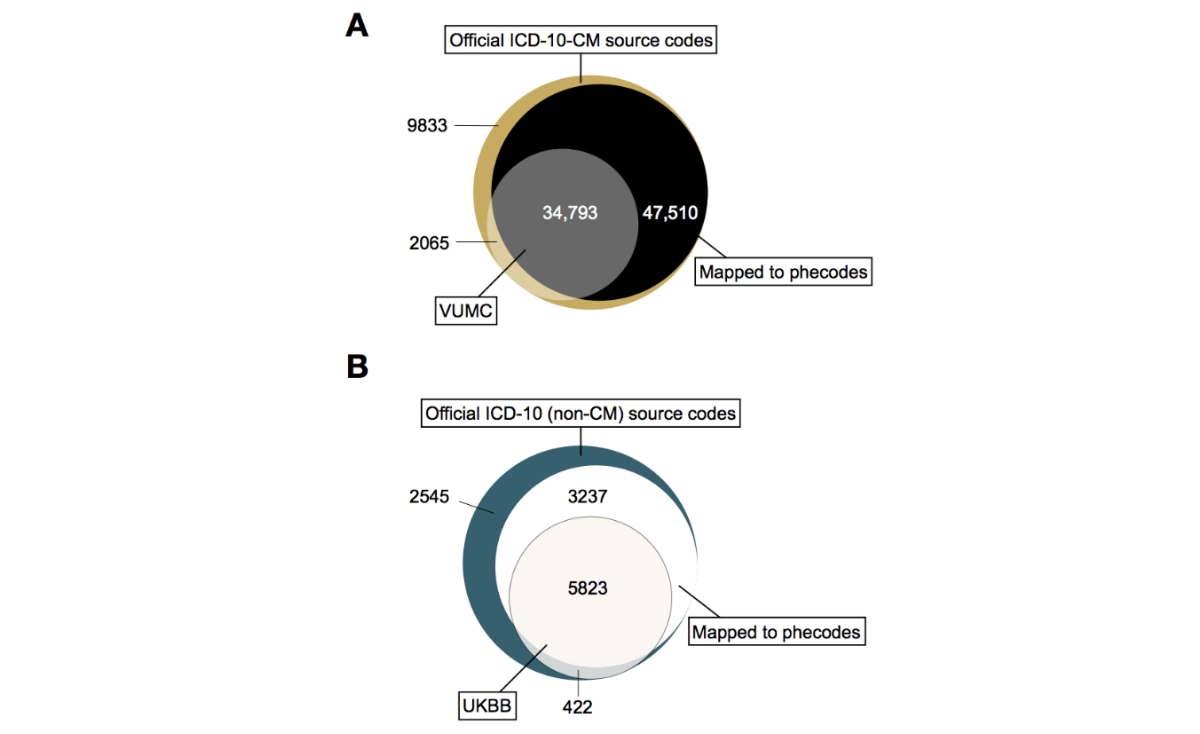
Counts of distinct ICD-10-CM source codes at VUMC and ICD-10 (non-CM) source codes in UKBB. (A) Number of unique ICD-10-CM codes in each category. For example, there were 34,793 unique codes (grey section) that were in the official ICD-10-CM system, observed in the VUMC dataset, and mapped to phecodes. (B) Number of unique ICD-10 codes in each category. For example, there were 5823 unique codes (off-white section) that were in the official ICD-10 system, observed in the UKBB dataset, and mapped to phecodes. ICD-10-CM: International Classification of Diseases, 10th Revision, Clinical Modification; VUMC: Vanderbilt University Medical Center; ICD-10: International Classification of Diseases, 10th Revision; UKBB: UK Biobank.

To evaluate the phecode coverage of ICD-10 and ICD-10-CM source codes in UKBB and VUMC, respectively, we calculated the number of source codes in the 2018AA UMLS, the number of source codes mapped to phecodes, and the number of mapped and unmapped source codes that were used in the two EHRs (Figure 2). To identify potential limitations of our automated mapping approach, two authors with clinical training (PW, WQW) manually reviewed all the unmapped ICD-10 and ICD-10-CM codes that were used at UKBB and VUMC, respectively.

### Comparison of Phenotypes Generated from the ICD-10-CM Phecode Map 

**Figure 3:**
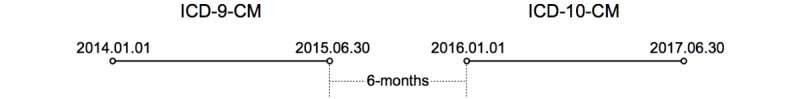
Timeline of the two 18-month periods from which ICD-9-CM and ICD-10-CM codes from VUMC were analyzed. The cohort of 357,728 patients had at least one ICD-9-CM and one ICD-10-CM code in the respective 18-month windows. ICD-9-CM: International Classification of Diseases, Ninth Revision, Clinical Modification; ICD-10-CM: International Classification of Diseases, 10th Revision, Clinical Modification.

We aimed to provide evidence that the ICD-10-CM phecode map resulted in phenotypes like those sourced from the ICD-9-CM phecode map. First, we selected 357,728 patients in the VUMC EHR who had ≥1 ICD-9-CM and ≥1 ICD-10-CM codes in two 18-month windows. We selected windows to occur prior to and after VUMC’s transition to ICD-10-CM. To reduce potential confounders, we left a 6-month buffer after ICD-9-CM was replaced with ICD-10-CM. Further, the ICD-10-CM observation window ended before VUMC switched from its locally developed EHR [27] to the Epic system. This created two windows ranging from January 1, 2014 to June 30, 2015 for ICD-9-CM, and January 1, 2016 to June 30, 2017 for ICD-10-CM (Figure 3). The final cohort consisted of 55.10% (197,109/357,728) females with mean age of 45 (SD 25) years old. From the two observation periods, we extracted all ICD-9-CM and ICD-10-CM codes for each patient. We then mapped these codes to phecodes using the ICD-9-CM phecode [14] and ICD-10-CM phecode maps.

We used the patient cohort to test our hypothesis that the ICD-10-CM phecode map created phenotype definitions that were comparable to those generated using the gold-standard ICD-9-CM phecode map. For this analysis, we used four common chronic diseases (Hypertension, Hyperlipidemia, Type 1 Diabetes, and Type 2 Diabetes) and chose one acute disease (Intestinal infection) as a negative control. We expected that a large majority of the chronic disease patients and a small minority of the acute disease patients from the ICD-9-CM era would reproduce the same phenotypes during the ICD-10-CM era. We defined the phenotype cases as follows: Hypertension with phecodes 401.* (* means one or more digits or a period); Hyperlipidemia, phecodes 272.*; Type 1 diabetes, phecodes 250.1*; Type 2 diabetes, phecodes 250.2*; Intestinal infection, phecodes 008.*.

For each phenotype, we reported the number of ICD-9-CM cases and the number of those individuals who were also ICD-10-CM cases. To identify the possible reasons for individuals who were not identified as phenotype cases in the ICD-10-CM period, two authors with clinical training (PW, WQW) manually reviewed the EHRs of ten randomly selected patients from each chronic disease group, except Type 1 diabetes, for a total of thirty patients.

### Comparative PheWAS Analysis of a Lipoprotein(a) Single-Nucleotide Polymorphism

To evaluate the accuracy of the ICD-10-CM phecode map, we performed two PheWASs on a Lipoprotein(a) (LPA) genetic variant (rs10455872) using mapped phecodes from ICD-9-CM and ICD-10-CM. The LPA single-nucleotide polymorphism (SNP) is associated with increased risks of developing hyperlipidemia and cardiovascular diseases [28-30].

We used data from BioVU, the deidentified DNA biobank at VUMC, to conduct the PheWAS [31]. We identified 13,900 adults (56.9% female; mean 59 [SD 15] years old in 2014), who had rs10455872 genotyped and at least one ICD-9-CM and ICD-10-CM code in their respective time windows. For rs10455872, we observed 86.7% AA, 12.8% AG, and 0.5% GG. We used 1632 phecodes that overlapped in the time windows for PheWAS using the R PheWAS package [13] with binary logistic regression, adjusting for age, sex, and race.

## Results

### Phecode Coverage of ICD-10-CM and ICD-10 in VUMC and UKBB

Of all possible ICD-10-CM codes [18], 82,303 (87.37%) mapped to at least one phecode, with 7881 (8.37%) mapping to >1 phecode. For example, ICD-10-CM I25.708 “Atherosclerosis of coronary artery bypass graft(s), unspecified, with other forms of angina pectoris” mapped to phecodes 411.3 “Angina pectoris” and 411.4 “Coronary atherosclerosis”. Of all possible ICD-10 codes, 9060 (75.33%) mapped to at least one phecode, and 289 (2.40%) mapped to >1 phecode. For example, ICD-10 code B21.1 “HIV disease resulting in Burkitt lymphoma” mapped to phecodes 071.1 “HIV infection, symptomatic” and 202.2 “Non-Hodgkins lymphoma”.

Among the 36,858 ICD-10-CM codes used at VUMC, 34,793 (94.40%) codes were mapped to phecodes. Of the 6245 ICD-10 codes used in the UKBB, 5823 (93.24%) codes mapped to phecodes (Table 1, Figure 2). Considering all the instances of ICD-10-CM and ICD-10 codes used at each site, we generated a total count of unique codes grouped by patient, date, and those codes that mapped to phecodes (Table 1). Among the total number of codes used, the vast majority of ICD-10-CM (17,658,470/19,682,697; 89.72%) and ICD-10 (4,279,544/5,114,363; 83.68%) codes were mapped to phecodes.

**Table 1:** ICD-10-CM and ICD-10 codes data summary.

		ICD-10-CM^a^ (VUMC^b^)	ICD-10^c^ (UKBB^d^)
**Official classification systems**			
	Unique codes, n	94,201	12,027
	Unique codes mapped, n (%)	82,303 (87.37)	9,060 (75.33)
**Official codes used in cohorts**			
	Unique codes, n	36,858	6,245
	Unique codes mapped, n (%)	34,793 (94.40)	5,823 (93.24)
Total patients (with ICD-10-CM or ICD-10 codes), n		651,649	391,181
Total instances of all ICD^e^ codes, n		19,682,697	5,114,363
Instances mapped to phecodes, n (%)		17,658,470 (89.72)	4,279,544 (83.68)

^a^ICD-10-CM: International Classification of Diseases, 10th Revision, Clinical Modification

^b^VUMC: Vanderbilt University Medical Center

^c^ICD-10: International Classification of Diseases, 10th Revision

^d^UKBB: UK Biobank

^e^ICD: International Classification of Diseases

### Analysis of Unmapped ICD-10 and ICD-10-CM Codes

Many of the unmapped ICD-10 codes used in the UKBB dataset represented medical concepts related to personal (ie, past medical history) or family history of disease. For ICD-10-CM, removing codes used at VUMC that we expected to be unmapped (ie, local or supplementary classification codes) left 2065 ICD-10-CM codes that did not map to a phecode. After excluding 1395 codes (eg, X, Y, and Z codes) indicating nonbiological disease phenotypes, 670 codes remained, the majority of which represented either external causes of morbidity or factors influencing health status and contact with health services. All the remaining unmapped ICD-10-CM codes in this cohort had <200 unique individuals (ie, <0.1% of the cohort), and the majority of the ICD-10-CM codes with >10 unique individuals were phenotypes that are most likely due to nongenetic factors. For example, 287 (59.2%) of the unmapped ICD-10-CM codes represented external causes of morbidity, such as assault and injuries due to motor vehicle accidents.

### Reproducibility Analysis of the ICD-10-CM Phecode map

In the defined 18-month time windows, a cohort of 357,728 patients had both ICD-9-CM and ICD-10-CM codes (Figure 3). For the chronic diseases, 70-75% of individuals with the relevant phecodes in the ICD-9-CM observation period also had the same phecodes of interest during the ICD-10-CM period. On the contrary, for the reproducibility analysis with an acute disease we observed that <10% of individuals who had phecodes 008.* (Intestinal infection) in the ICD-9-CM period also had the same phecodes in the ICD-10-CM period (Table 2).

To identify the reasons that may explain why some patients were not identified as cases for the phenotype of interest during the ICD-10-CM period, we manually reviewed their medical records. A total of 30 patients were selected for review, 10 each from the Hypertension, Hyperlipidemia, and Type 2 diabetes cohorts (see Multimedia Appendix 1). We found that none of the patients had a relevant ICD-10-CM code for the phenotype being studied in the 18-month observation period. Reasons for patients not being ICD-10-CM cases included: patients were labeled with the relevant ICD-10-CM code(s) outside of the short ICD-10-CM observation window (8 patients), patients had <2 visits at VUMC during the ICD-10-CM period or were only seen by physician specialists (10 patients; eg, a patient with hypertension was only seen by their neurologist during the ICD-10-CM period), and patients were inconsistently diagnosed (2 people; eg, patient with Type 1 diabetes given Type 2 diabetes ICD-9-CM code). No cases were missed due to errors in the ICD-10-CM phecode map.

**Table 2:** ICD-10-CM phecode map reproducibility analysis.

Phenotype	Phecodes^a^	ICD-9-CM^b^ cases (n)	ICD-10-CM^c^ case|ICD-9-CM case^d^, n (%)
Hypertension	401.*	65,216	49,468 (75.85)
Hyperlipidemia	272.*	51,187	36,187 (70.7)
Type 1 diabetes	250.1*	5782	4412 (76.31)
Type 2 diabetes	250.2*	25,077	19,066 (76.03)
Intestinal infection	008.*	3410	273 (8.01)

^a^In the phecode column, * means ≥1 digits or a period (eg, phecode 401.*=phecodes 401, 401.1, 401.3, 401.22, 401.21, or 401.2)

^b^ICD-9-CM: International Classification of Diseases, Ninth Revision, Clinical Modification

^c^ICD-10-CM: International Classification of Diseases, 10th Revision, Clinical Modification

^d^In the last column, “ICD-10-CM case|ICD-9-CM case” indicates patients who were cases for the phenotype of interest during the ICD-9-CM period who were also ICD-10-CM cases

### Comparative PheWAS Analysis of the Lipoprotein(a) SNP, rs10455872

**Figure 4:**
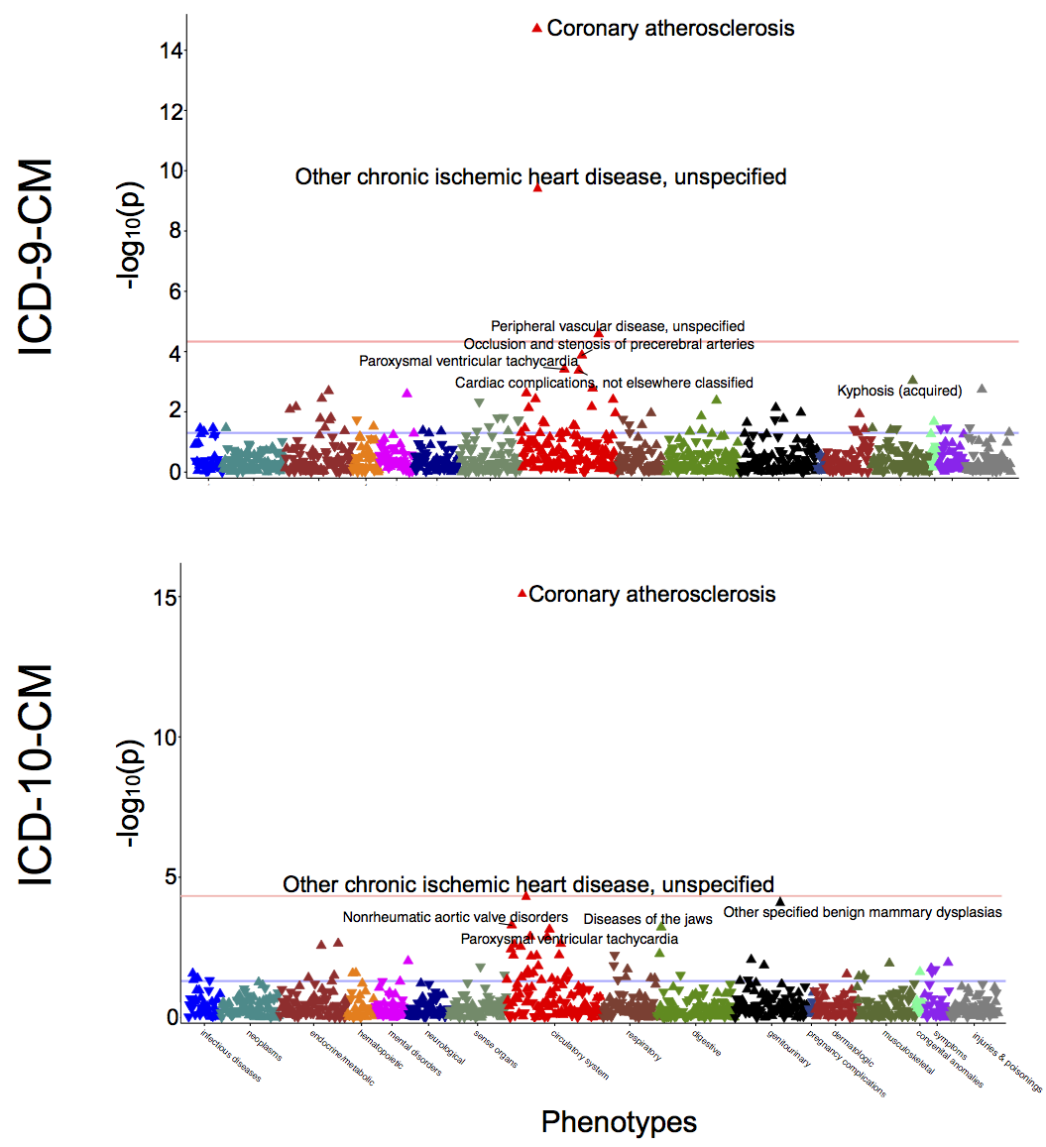
Comparative PheWAS of lipoprotein(a) genetic variant, rs10455872. “Coronary atherosclerosis” (phecode 411.4) and “Other chronic ischemic heart disease” (phecode 411.8) were top hits associated with rs10455872 in a PheWAS analysis conducted using ICD-9-CM (top) and ICD-10-CM (bottom) phecode maps. Analyses were adjusted for age, sex, and race. PheWAS: phenome-wide association studies; ICD-9-CM: International Classification of Diseases, Ninth Revision, Clinical Modification; ICD-10-CM: International Classification of Diseases, 10th Revision, Clinical Modification.

To further evaluate the ICD-10-CM phecode map, we performed and compared the results of PheWAS analyses for rs10455872. One PheWAS was conducted using the ICD-9-CM map and another was conducted using the ICD-10-CM map. Both analyses replicated previous findings with similar effect sizes: coronary atherosclerosis (ICD-9-CM: *P*<.001; odds ratio [OR] 1.60 [95% CI 1.43-1.80] vs ICD-10-CM: *P*<.001, OR 1.60 [95% CI 1.43-1.80]) and chronic ischemic heart disease (ICD-9-CM: *P*<.001; OR 1.56, [95% CI 1.35-1.79] vs ICD-10-CM: *P*<.001, OR 1.47 [95% CI 1.22-1.77]) (Figure 4).

## Discussion

### Maps of ICD-10 and ICD-10-CM Codes to Phecodes have High Coverage and Yield Similar Results

In this study, we described the process of mapping ICD-10 and ICD-10-CM codes to phecodes and evaluated the results of the new maps in two databases. These results show that the majority of the ICD-10 and ICD-10-CM codes used in EHRs were mapped to phecodes. Our analyses suggest that researchers can expect that phenotypes sourced using the ICD-10-CM phecode map will be like those sourced from the gold-standard ICD-9-CM phecode map. As the use of ICD-10 and ICD-10-CM codes increases, so does the need for convenient and reliable methods of aggregating codes to represent clinically meaningful phenotypes. 

Since the introduction of phecodes, many studies have demonstrated the value of aggregating ICD-9-CM codes for genetic association studies. These maps will allow biomedical researchers to leverage clinical data represented by ICD-10 and ICD-10-CM codes for their large-scale PheWAS using EHRs. They will also allow researchers to combine phenotypes as phecodes mapped from ICD-9- and ICD-10-based coding systems, thereby increasing the size of their patient cohorts and statistical power of their studies. The maps are available from the PheWAS Resources page [14] and are incorporated in the PheWAS R package, version 0.99.5-2 [13,32]. 

### ICD-10 and ICD-10-CM Codes not Mapped to Phecodes

Analysis of the unmapped ICD-10 codes demonstrates a possible area of expansion for phecodes. The ICD-10 phecode map did not include medical concepts representing personal history or family history of disease.

We observed that a majority of the unmapped ICD-10-CM codes represented concepts that we did not expect to have phecode equivalents. Most of the codes were from ICD-10-CM chapters 20, “External causes of morbidity” and 21, “Factors influencing health status and contact with health services”. Codes from chapter 19, “Injury, poisoning, and certain other consequences of external causes” also made up a large proportion of unmapped codes, such as ICD-10-CM T38.3X6A, “Underdosing of insulin and oral hypoglycemic [antidiabetic] drugs, initial encounter”. We did not expect ICD-10-CM T38.3X6A to map to a phecode, as it is an encounter code that is not relevant to PheWAS. Three-digit codes that are not frequently used for reimbursement purposes, such as ICD-10-CM I67, “Other cerebrovascular diseases”, also made up many unmapped codes. A few potential clinically meaningful phenotypes, such as ICD-10-CM O04.6, “Delayed or excessive hemorrhage following [induced] termination of pregnancy”, were unmapped and represent areas of potential expansion for phecodes.

### ICD-10-CM Phecode Map Phenotype Reproducibility Analysis

In general, our analysis suggests that in most of the cases in which phenotypes are not reproduced in the ICD-10-CM observation period, they are not due to errors in the ICD-10-CM phecode map.. This study’s reproducibility analysis (Table 2) demonstrates that most patients (70-75%) with phecodes of four chronic diseases sourced from ICD-9-CM codes were also phenotype cases in the ICD-10-CM era. In comparison, when the same experiment is repeated for an acute disease (Intestinal infection), a minority (<10%) of patients had the same phenotype in the ICD-10-CM period. 

Using the ICD-9-CM and ICD-10-CM maps, PheWAS found significant genetic associations with similar effect sizes for coronary atherosclerosis and chronic ischemic heart disease (Figure 4). Results of this analysis provide additional support for the accuracy of the ICD-10-CM map when compared to the gold-standard ICD-9-CM phecode map.

### PheWAS Using ICD-10 Phecode Map

Two published studies have used the ICD-10 phecode map to identify genotype-phenotype associations using UKBB data. Zhou et al used the map to demonstrate a method that adjusts for case-control imbalances in a large genome-wide PheWAS [33], and Li et al used the same map to estimate the causal effects of elevated serum uric acid across the phenome [12].

### Utilization of Phecodes Outside of PheWAS

In addition to being employed for PheWAS, phecodes have been used to answer a range of questions in biomedicine. Phecodes have been used to identify features in radiographic images that are associated with disease phenotypes [34] and used in machine learning models to improve cardiovascular disease prediction [35]. In a recent study to understand public opinion about diseases, Huang et al identified articles about diseases and mapped them to phecodes [36]. Motivated by the difficulties in automatically translating diagnosis codes from EHRs, Shi et al used phecodes to map ICD-9-CM diagnosis codes from one health system to another [37]. Phecodes have also been applied to identify conditions for aggregation in phenotype risk scores, much as SNPs are aggregated as a genetic risk score to identify Mendelian diseases and determine pathogenicity of genetic variants [38].

### Related Work

The Clinical Classification Software (CCS) is another maintained system for aggregating ICD codes into clinically meaningful phenotypes. CCS was originally developed by the Agency for Healthcare Research and Quality (AHRQ) to cluster ICD-9-CM diagnosis and procedure codes to a smaller number of clinically meaningful categories [39]. CCS has been used for many purposes, such as measuring outcomes [40] and predicting future health care usage [41]. In a previous study, we showed that phecodes were better aligned with diseases mentioned in clinical practice and that were relevant to genomic studies than CCS for ICD-9-CM (CCS9) codes [20]. We found that phecodes outperform CCS9 codes, in part because CCS9 was not as granular as phecodes. Since CCS for ICD-10-CM (CCS10) is of similar granularity as CCS9 (283 versus 285 disease groups) [42], we believe that the phecode map would likely still better represent clinically meaningful phenotypes in genetic research.

### Limitations

This study has limitations. First, only 84.14% (1570/1866) of phecodes are mapped to at least one ICD-10 code. This may be due in part to the automated strategy that we used to map ICD-10 to ICD-9-CM. Second, the VUMC data are from a single site, thereby making it difficult to generalize the results of our accuracy studies (eg, phenotype reproducibility analysis and LPA SNP PheWAS) to patient cohorts in other EHRs. Third, we have not yet manually reviewed all the mappings in these beta phecode maps, and our assumptions that the manually reviewed resources (eg, NLM and OHDSI) are highly accurate could have affected the accuracy of the new phecode maps. For example, in the 2009 ICD-10-CM to ICD-9-CM GEMS, >90% of the mappings were approximate (ie, nonequivalent) [15]. For this study’s purposes, we aimed to maximize phecode coverage of ICD source codes and thus included both equivalent and nonequivalent 2018 GEMS translations, which could have decreased mapping performance.

Fourth, our automated approach to map >80,000 ICD-10-CM and >9000 ICD-10 codes to phecodes with minimal human engineering could have decreased the accuracy of the final maps. Hripcsak et al [43] recently evaluated the effects of translating ICD-9-CM codes to SNOMED CT codes on the creation of patient cohorts. In general, they found that mapping source billing codes to a standard clinical vocabulary (eg, ICD-9-CM to SNOMED CT) did not greatly affect cohort selection. Their findings suggested that optimized domain knowledge–engineered mappings outperformed simple automated translations between clinical vocabularies. Using four phenotype concept sets, they showed that automated mappings resulted in errors of up to 10% and that domain-knowledge engineered mappings had errors of <0.5%. Other studies have also found that mapping performance is generally better with smaller value sets [17]. To create a more comprehensive and accurate map between ICD-9-CM and ICD-10-CM, future mapping studies could consider using an iterative forward and backward mapping approach using GEMS [17]. 

### Future Directions

Currently, if an ICD-10 or ICD-10-CM code maps to ≥2 unlinked phecodes, we keep all the mappings. In subsequent studies, it will be important to further scrutinize these mappings to ensure accuracy through manual review. As new ICD-10-CM codes are released, we plan to assess their relevance to clinical practice and genetic research and decide whether we should translate them to phecodes. We intend to address the unmapped source codes (eg, ICD-10-CM E78.41 “Elevated Lipoprotein(a)”) by potentially expanding the phecode system, and to systematically evaluate the mappings with input from users. 

### Conclusions

In this paper, we introduced our work on mapping ICD-10 and ICD-10-CM codes to phecodes. We provide initial beta maps with high coverage of EHR data in two large databases. Results from this study suggested that the ICD-10-CM phecode map created phenotypes similar to those generated by the ICD-9-CM phecode map. These mappings will enable researchers to leverage accumulated ICD-10 and ICD-10-CM data in the EHR for large PheWAS.

## Appendix

Multimedia Appendix 1 ICD-10-CM reproducibility analysis, manual chart review results.

## Abbreviations

AHRQ: Agency for Healthcare Research and Quality
CCS: Clinical Classification Software
CUI: Concept Unique Identifier
EHR: electronic health record
GEMS: General Equivalence Mappings
ICD: International Classification of Diseases
ICD-9-CM: International Classification of Diseases, Ninth Revision, Clinical Modification
ICD-10: International Classification of Diseases, 10th Revision
ICD-10-CM: International Classification of Diseases, 10th Revision, Clinical Modification
LPA: lipoprotein(a)
NCHS: National Center for Health Statistics
NLM: National Library of Medicine
OHDSI: Observational Health Data Sciences and Informatics
OR: odds ratio
PheWAS: phenome-wide association studies
SNOMED CT: Systematized Nomenclature of Medicine Clinical Terms
SNP: single nucleotide polymorphism
UKBB: UK Biobank
UMLS: Unified Medical Language System
VUMC: Vanderbilt University Medical Center
WHO: World Health Organization
